# How Malaria Parasites Acquire Nutrients From Their Host

**DOI:** 10.3389/fcell.2021.649184

**Published:** 2021-03-25

**Authors:** Natalie A. Counihan, Joyanta K. Modak, Tania F. de Koning-Ward

**Affiliations:** School of Medicine, Deakin University, Waurn Ponds, VIC, Australia

**Keywords:** *Plasmodium*, malaria, nutrients, new permeation pathway, transporters

## Abstract

*Plasmodium* parasites responsible for the disease malaria reside within erythrocytes. Inside this niche host cell, parasites internalize and digest host hemoglobin to source amino acids required for protein production. However, hemoglobin does not contain isoleucine, an amino acid essential for *Plasmodium* growth, and the parasite cannot synthesize it *de novo*. The parasite is also more metabolically active than its host cell, and the rate at which some nutrients are consumed exceeds the rate at which they can be taken up by erythrocyte transporters. To overcome these constraints, *Plasmodium* parasites increase the permeability of the erythrocyte membrane to isoleucine and other low-molecular-weight solutes it requires for growth by forming new permeation pathways (NPPs). In addition to the erythrocyte membrane, host nutrients also need to cross the encasing parasitophorous vacuole membrane (PVM) and the parasite plasma membrane to access the parasite. This review outlines recent advances that have been made in identifying the molecular constituents of the NPPs, the PVM nutrient channel, and the endocytic apparatus that transports host hemoglobin and identifies key knowledge gaps that remain. Importantly, blocking the ability of *Plasmodium* to source essential nutrients is lethal to the parasite, and thus, components of these key pathways represent potential antimalaria drug targets.

## Introduction

**FIGURE 1:**
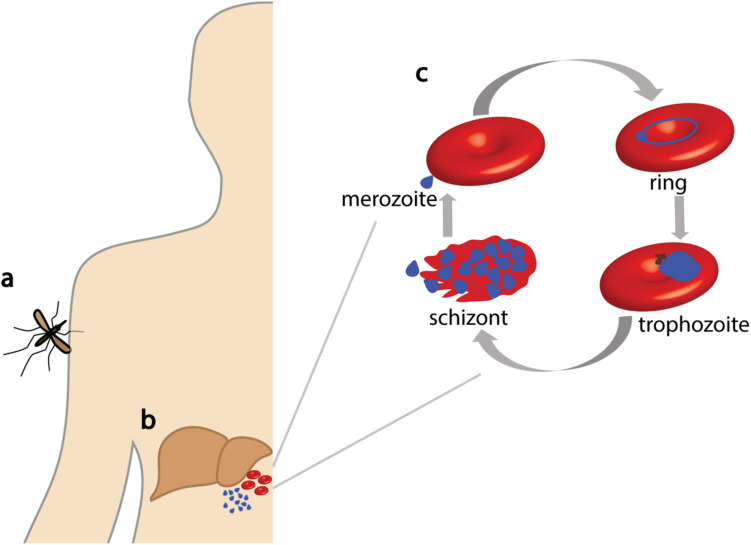
Lifecycle of *Plasmodium falciparum*. *P. falciparum* parasites are injected into a human host via mosquito bites **(a)** where they then travel to the liver **(b)**. After parasite replication, merozoites are released into the blood where they invade erythrocytes to commence the asexual replication cycle **(c)**. Parasites develop within the erythrocyte in distinct forms (ring, trophozoite, and schizont) until erythrocyte lysis occurs, releasing merozoites for reinvasion.

Malaria is a devastating infectious disease caused by protozoan parasites belonging to the species *Plasmodium*, with an estimated 228 million cases in 2018 alone (World Health Organization, 2019). Despite a modest reduction in the burden of malaria in the last 20 years, it remains a global health problem because of the absence of an effective vaccine and limited chemotherapeutic options as a result of drug resistance. The most severe form of malaria is caused by *Plasmodium falciparum* that is transmitted between people via a mosquito vector (Figure 1a). After infection, the parasite has a complex lifecycle that includes an asymptomatic liver stage (Figure 1b). Following replication inside hepatocytes, merozoites are released into the blood where they invade erythrocytes to initiate the blood stage of the parasite lifecycle; here, rapid asexual replication occurs, and the clinical symptoms of malaria become evident (Figure 1c).

*Plasmodium falciparum* is an obligate intracellular parasite and is dependent on its host to supply the nutrients required to support its development. However, *P. falciparum* faces some challenges by selecting to reside in mature, metabolically inactive erythrocytes. While the parasites are able to take up hemoglobin from the host cell cytoplasm and rapidly metabolize it, human hemoglobin does not contain all the amino acids necessary for *P. falciparum* growth; isoleucine is absent, and methionine, for example, is poorly represented, and thus these amino acids must be acquired from the human serum (Sherman, 1977; Divo et al., 1985; Spry et al., 2010). So too does pantothenate (vitamin B_5_), the precursor to coenzyme A, as it cannot be synthesized by the parasite *de novo*, and it is also essential for parasite growth (Saliba et al., 1998). Although *P. falciparum* can synthesize thiamine (vitamin B_1_), it cannot do so in sufficient quantities (Wrenger et al., 2006). Also, rate-limiting to parasite growth are the diminishing intracellular pools of other nutrients including folate and purines (Liu et al., 2006; Wang et al., 2007), and as *P. falciparum* is unable to synthesize purines, it is dependent upon the salvage of exogenous purines via the purine salvage pathway (Reyes et al., 1982; Downie et al., 2008). While *P. falciparum* has some capacity to synthesize its own lipids (reviewed in O’Neal et al., 2020), several classes of lipids are rate-limiting and must also be obtained from an extracellular source (Gulati et al., 2015; O’Neal et al., 2020).

**FIGURE 2:**
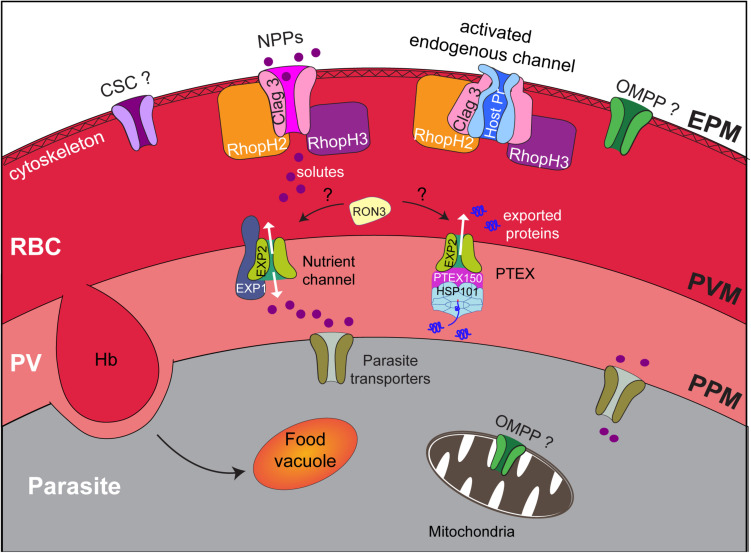
Nutrient acquisition by *P. falciparum* relies on membrane transporters and the cytostome. Upon infection by *Plasmodium* parasites, erythrocytes are extensively remodeled to accommodate the growing parasite. The parasite, contained by its own PPM, is encased within a parasitophorous vacuole (PV) and a PV membrane (PVM). Each membrane in the infected erythrocyte, including the erythrocyte plasma membrane (EPM), is modified to include transporters that enable the parasite to access nutrients from its host. These include the NPPs at the EPM, the nutrient channel at the PVM, and transporters at the PPM, whereas hemoglobin uptake and transport to the food vacuole occur via the cytostome. The NPP channel is proposed to be a CLAG3 dimer/oligomer that associates with RhopH2 and RhopH3. Alternatively, CLAG3, RhopH2, and RhopH3 may activate an endogenous host channel. The contribution of other parasite-encoded channels to the NPPs such as the calcium-dependent, stress-gated ion channel (CSC), and OMPP is unknown; the localization of both of these proteins is yet to be ascertained. The nutrient channel at the PVM comprises EXP2, with EXP1 being critical for proper distribution of the EXP2 nutrient channel. EXP2 also forms the pore of the translocon of exported proteins (PTEX). RON3 is implicated in both nutrient uptake across the PVM and protein export with an unknown function. The PPM is also decorated with a raft of transporters that facilitate the uptake of diverse substrates to satisfy the metabolic requirements of the growing parasite.

As *P. falciparum* resides within a parasitophorous vacuole (PV) in the host erythrocyte (Figure 2), nutrients acquired from the extracellular milieu must traverse otherwise impermeable membranes, namely, the erythrocyte plasma membrane (EPM), the PV membrane (PVM), and the parasite plasma membrane (PPM). The erythrocyte plasma membrane harbors a range of specialized transporters that form channels, carriers, or pumps to facilitate the passage of substrates. However, not all substrates required by *Plasmodium* (e.g., isoleucine and pantothenate) can be obtained via the endogenous transporters, or they are transported in insufficient quantity (Saliba et al., 1998; Martin and Kirk, 2007). Therefore, additional mechanisms must be employed to enable *P. falciparum* to acquire essential nutrients from the host serum. This is achieved through the creation of new permeation pathways (NPPs) that facilitate the transport of a broad range of substrates across the erythrocyte membrane (Ginsburg et al., 1985; Desai et al., 1993). NPPs result from modifications to the host erythrocyte membrane (Figure 2); they behave as channels and display a preference for anions over cations (Kirk et al., 1994), but also likely act to dispel waste products generated from hemoglobin digestion and metabolic processes and to maintain ion gradients essential for cell function. NPPs are established at the erythrocyte membrane from 15 h after invasion and reach a plateau after 36 h (Staines et al., 2001). Despite the importance of this channel to parasite survival and its potential as a drug target, until recently, very little was known about its molecular composition, especially compared with our current knowledge of other *P. falciparum* transporters (reviewed in Martin, 2020). The molecular makeup of the “sieve” that allows host nutrients to then cross the encasing PVM so that they can then be transported by the many transporters that decorate the PPM has also only just been revealed. So too have components of the endocytic apparatus that traffics hemoglobin to the digestive food vacuole. This review discusses the strategies the parasite employs to obtain nutrients from the host serum and host cell cytoplasm and the recent advances that have been made in identifying the molecular constituents of these pathways.

## Crossing the Erythrocyte Membrane

The human erythrocyte membrane contains more than 50 types of transmembrane proteins in its lipid bilayer that are responsible for a range of functions including substrate transport, cell adhesion, and structural integrity (reviewed in Pretini et al., 2019). Substrate transport is facilitated by ATP-powered transporters, organic cation and anion channels, glucose transporters, *P*-type ATPases, urea transporters, and aquaporins, as well as monocarboxylate transporter 1 (MCP1), anion-exchanger 1, and equilibrative nucleoside transporter 1 (Pretini et al., 2019). These transporters serve to maintain the selective permeability of the membrane. It has long been recognized that *P. falciparum* infection induces a measurable increase in the permeability of host erythrocytes (Overman, 1947) to a diverse range of structurally unrelated low molecular mass solutes including sugars, amino acids, vitamins, purines, and ions (reviewed in Martin, 2020), and this change is attributed to NPPs (Ginsburg et al., 1985; Kirk et al., 1994). As the NPPs have the characteristics of an anion-selective channel, it is referred to as the *Plasmodium* surface anion channel (PSAC) (reviewed in Desai, 2012). Despite the recognition of the existence of NPPs for several decades, its biochemical composition has not been well defined, and indeed, it is not known whether PSAC represents the only NPP and whether the NPPs are solely parasite-derived or comprise host cell constituents.

### Contribution of CLAG Proteins to NPPs

The *P. falciparum* gene products encoded by *clag3.1* (PF3D7_0302500) and *clag3.2* (PF3D7_0302200) genes were the first parasite-encoded proteins implicated in NPPs and were identified using nutrient restriction, NPP inhibitors, and genetic approaches (Nguitragool et al., 2011; Pillai et al., 2012; Figure 2). Parasites in which CLAG3 expression has been depleted through either epigenetic silencing or *in vitro* selection exhibit delayed *in vitro* growth (Comeaux et al., 2011) and altered PSAC activity (Mira-Martinez et al., 2013; Sharma et al., 2013, 2015); yet, interestingly, parasites are still able to survive. Even complete knockout of the *clag3* genes is not lethal, and transport activity is not completely lost in these parasites (Gupta et al., 2020). In the latter case, parasites only displayed a fitness cost when grown in a modified medium that more closely resembles human plasma.

That the CLAG3 proteins were implicated in NPPs was surprising as they do not resemble known transporters, and moreover, it was expected that proteins comprising the NPP channel would be essential for parasite survival given the vital nature of the substrates it transports. However, *clag3.1* and *clag3.2* belong to a unique *Plasmodium* multigene family referred to as RhopH1, which also comprises *clag2*, *clag8*, and *clag9* in *P. falciparum* (Kaneko et al., 2001; Ling et al., 2004; Kaneko et al., 2005). As all five *clag* genes have the same intron–exon structure and are highly conserved, it is conceivable that the loss of the *clag3* genes can be functionally complemented by the other paralogs. Unfortunately, this is very difficult to assess in *P. falciparum* given the technical limitations of knocking out multiple *clag* paralogs in the one parasite line. Alternatively, it is possible that the different *clag* paralogs form distinct channels that facilitate the uptake of different solutes. To date, gene knockouts of *clag2* and *clag8* have not been reported in the literature to assess this. However, *clag2* expression was found to be silenced in blasticidin-resistant parasites in which silencing of both *clag3* genes led to reduced uptake of blasticidin (Sharma et al., 2013, 2015). This is despite *clag2* expression being independent of the epigenetically regulated *clag3* switching program (Cortés et al., 2007). This finding suggests that *clag2* may also contribute to NPP function. For *clag9*, several studies have shown that this gene is not essential for *P. falciparum* growth *in vitro.* Interestingly, CLAG9 is the most distinct of all the CLAG paralogs and lacks the small hypervariable region that is present in its paralogs and which has been shown to be exposed at the erythrocyte surface (Nguitragool et al., 2011, 2014). However, to date, no studies have directly examined the impact of the loss of *clag9* on solute uptake. Prior studies have instead linked CLAG9 to cytoadherence of infected erythrocytes to CS32 melanoma cells and the endothelial receptor CD36, potentially via assisting with the trafficking of the major virulence factor PfEMP1 onto the erythrocyte surface (Trenholme et al., 2000; Goel et al., 2010). However, a subsequent study observed that the loss of binding of infected erythrocytes to CD36 was a result of expression of a non-functional PfEMP1 rather than lack of *clag9* (Nacer et al., 2011). CLAG9 has also been linked to merozoite binding to erythrocytes by interacting with the glycophorin A–band3 receptor–co-receptor complex (Chourasia et al., 2020). Although it is not uncommon for protein and protein fragments to bind non-specifically to erythrocyte membranes, RhopH3 that forms a complex with *clag9* (discussed below) has also been shown to be involved in erythrocyte invasion. Nevertheless, at this stage, it still remains to be formally examined whether CLAG9 also plays a role in NPP activity.

### Contribution of RhopH2 and RhopH3 to NPP

The CLAG3 proteins are synthesized toward the end of the parasite asexual cell cycle, where they are stored in the rhoptry organelle in a complex, termed the RhopH complex, with two unrelated proteins named RhopH2 and RhopH3, which are encoded by single copy genes [(Cooper et al., 1988) and reviewed in Counihan et al. (2013)]. The RhopH protein complex is highly conserved among *Plasmodium* species, although the number of *Clag* genes varies among *Plasmodium* species. Upon merozoite invasion, all three proteins are transferred to the newly infected erythrocyte (Vincensini et al., 2008) and are transported to the host cell membrane via undefined trafficking routes. At the erythrocyte membrane, RhopH1, RhopH2, and RhopH3 exist as a complex (Figure 2; Sam-Yellowe et al., 1988) in a stoichiometry yet to be resolved, where RhopH2 has been shown to associate with host skeletal proteins (Counihan et al., 2017). Both the *rhopH2* and *rhopH3* genes are refractory to deletion and essential for *Plasmodium* survival (Cowman et al., 2000; Janse et al., 2011; Zhang et al., 2018). Using inducible knockdown/knockout approaches, depletion of RhopH2 and RhopH3 caused a significant defect in parasite growth, and NPP activity was limited (Counihan et al., 2017; Ito et al., 2017; Sherling et al., 2017). Interestingly, depletion of RhopH2 resulted in a reduction of essential vitamins and cofactors including folate and decreased *de novo* synthesis of pyrimidines, which likely contributed to the delayed growth phenotype observed (Counihan et al., 2017). Normally, *P. falciparum* requires ∼48 h to complete a lifecycle, and rapid growth and maturation are observed in the second half of the cycle (Figure 1); reduced levels of RhopH2 and RhopH3 resulted in parasites stalling about midway through their lifecycle, which is consistent with when NPPs are active (15–36 h after invasion).

### What Is the Channel-Forming Component of the NPP?

There is compelling evidence that the RhopH proteins participate in NPP formation, but there are still fundamental questions concerning the makeup of NPP that need to be determined, particularly the component(s) that form the actual channel through the erythrocyte membrane. The CLAG proteins do not resemble any known proteins, including known transporters, although some of the top hits to the CLAG3 proteins using the protein prediction and structure program iTasser (Zhang, 2008) are channel proteins. The CLAGs are predicted to harbor a region that may form a transmembrane domain that is lined with polar residues on one side and non-polar on the other, reminiscent of the domains that form water-filled pores (Sharma et al., 2015), and a pool of CLAG3 is integral to the membrane (Nguitragool et al., 2011).

A soluble form of RhopH protein complex structure has recently been solved via cryoelectron microscopy (Schureck et al., 2021). Structural analysis reveals a heterotrimeric complex of CLAG3, RhopH2, and RhopH3 with a 1:1:1 stoichiometry. CLAG3 is situated in the middle of the complex, where it interacts tightly with RhopH2 and RhopH3. However, the predicted transmembrane domain of CLAG3, which does indeed form an a-helix near the CLAG3 *C*-terminus, is buried in the core of the RhopH complex and is not exposed to solvent. So too is the hypervariable region that was previously shown to be exposed on the surface of infected erythrocytes (Nguitragool et al., 2011). Thus, in order for the complex to be incorporated into the membrane, large-scale conformational changes would be required. The process(es) that may drive this are unknown but could potentially involve posttranslational modifications such as phosphorylation or regulation of allosteric disulfides or may require interactions with proteins or lipids at the erythrocyte plasma membrane.

RhopH2 and RhopH3 are even less convincing as potential channel proteins, showing no structural homology to known channel proteins. Moreover, protease protection assays indicate that RhopH2 and RhopH3 are not exposed on the erythrocyte surface (Ito et al., 2017). Some protein prediction programs such as Phobius suggest the presence of at least 1 transmembrane domain in RhopH2 and RhopH3, and thus, there is the possibility that CLAG forms a functional channel with RhopH2 and RhopH3 or indeed forms homo-oligomers or oligomerizes with other proteins to form a channel (see Figure 2 for a model). However, the predicted transmembrane domains of RhopH2 and RhopH3 are also buried in the RhopH soluble structure, 46–101 Å apart from the CLAG3 predicted transmembrane domain. Thus, massive rearrangement of the complex structure would be required if the RhopH2, RhopH3, and CLAG3 transmembrane domains come together to form a channel in the erythrocyte plasma membrane (Schureck et al., 2021). Given the contribution of RhopH2 and RhopH3 to NPP function, it is likely, therefore, that these two proteins play an essential accessory role.

There is also the possibility that CLAG3 is not the NPP channel and that other parasite or host proteins serve this function (Figure 2). Parasite-encoded proteins that exhibit homology to anion-selective channels have been identified among the *Plasmodium* transportome, including the outer membrane pore-forming protein (OMPP) found in bacteria and eukaryotic organelles such as the mitochondria and chloroplasts (Reddy and Saier, 2016) and the calcium-dependent, stress-gated ion channel (CSC) that belongs to the calcium-dependent chloride channel (Ca-CIC) family (Martin, 2020). The localization of both of these proteins in *Plasmodium* has yet to be assessed; only CSC harbors a signal sequence, and neither protein is predicted to contain export motifs to facilitate trafficking into the erythrocyte after synthesis. Neither of these proteins interacts with members of the RhopH complex; thus, if they are involved in NPPs, they must function independently of the RhopH complex (Figure 2).

Immunoprecipitation experiments have revealed that ∼30 parasite proteins affinity purify with RhopH2 (Counihan et al., 2017). While these proteins are exported or predicted to be exported into the host cytoplasm, none resemble channel proteins, and therefore, unlikely to serve as the NPP channel. Interestingly, erythrocyte anion exchanger 1 (AE1) affinity is purified with the RhopH complex, but as this endogenous protein is highly abundant, its binding may be non-specific. Moreover, if AE1 served as the NPP channel, it would have to be modified by the parasite during infection given that NPPs are not established until 15 h postinfection. While AE1 is progressively phosphorylated in response to oxidative stress when parasites transition from the ring to schizont stage (Pantaleo et al., 2010), the resulting reduction in AE1 affinity for the cytoskeleton and consequential membrane destabilization occur at the end of the cell cycle (Ferru et al., 2011), which is more consistent with a role in merozoite egress rather than NPP function.

Another erythrocyte voltage-dependent anion channel (VDAC), which is either expressed alone or as a component of the peripheral-type benzodiazepine receptor (PBR) complex, is the VDAC. In uninfected erythrocytes, PBR mediates the AE1-independent anion conductance and becomes upregulated when erythrocytes are infected by *P. falciparum* (Bouyer et al., 2011). The properties of VDAC resemble those of the NPP, and PBR ligands reduce membrane transport and conductance in *P. falciparum-*infected erythrocytes and block parasite growth *in vitro.* However, VDAC/PBR does not immunoprecipitate with the RhopH complex (Counihan et al., 2017), indicating that this transporter exists as an independent entity at the erythrocyte membrane and is not regulated by the RhopH components, but whether it associates with other parasite proteins is unknown. It also remains unknown what substrates VDAC/PBR transports in *Plasmodium-*infected erythrocytes. Thus, despite the importance of NPPs to parasite growth, the molecular composition of this channel still remains elusive.

## Nutrient Transfer Across the PVM

The erythrocyte membrane is not the only barrier to host nutrient uptake by the parasite. Nutrients must also be able to cross the PVM. Soluble macromolecules such as amino acids and monosaccharides up to 1,400 Da in size are able to readily cross this membrane via a channel that is permeable to both cations and anions (Desai and Rosenberg, 1997). Cell-attached patch clamping on parasites isolated from infected erythrocytes has revealed that this channel is present at the PVM in high density and is open most of the time (Desai et al., 1993). Permeation through this channel does not involve the binding of substrates; rather, the channel behaves like a sieve. Thus, transfer across the PVM is constrained by size of the solute and its diffusion coefficient in the cytosol (Desai et al., 1993; Desai and Rosenberg, 1997).

The PVM in the related apicomplexan parasite, *Toxoplasma gondii*, has a similar exclusion limit for macromolecules to that of *Plasmodium* (Schwab et al., 1994), suggesting that related channels may exist to transport soluble macromolecules across their respective PVMs. Two *T. gondii* proteins, termed GRA17 and GRA23, which are secreted from the dense granule organelles following invasion, perform this role. These proteins were shown to be required for the uptake of membrane-impermeable dyes lucifer yellow (522 Da) and the fluorescent form of 5-(and-6)-carboxy-20,70-dichlorofluorescein diacetate (CDCFDA; 445 Da) into the PV (Gold et al., 2015). In contrast, and consistent with the size constraint of this channel, dextran (3,000 Da) was excluded from entry. Moreover, injection of GRA17 and GRA23 cRNA into *Xenopus* oocytes altered their membrane conductance (Gold et al., 2015). Although the GRA proteins appear to act synergistically, it remains unclear if they have separate or overlapping functions. Studies in mice have revealed that GRA17-mediated PVM permeability is important for the growth of tachyzoites (the rapidly growing life stage of *T. gondii*) and virulence in mice, whereas both GRA17 and GRA23 are important for the viability of the *T. gondii* life stage inside cysts (Gold et al., 2015; Paredes-Santos et al., 2019; Li et al., 2020).

Interestingly, GRA17 and GRA23 show predicted structural similarity to *Plasmodium* EXP2, the pore-forming component of the *Plasmodium* translocon of exported proteins (PTEX) that is also secreted from dense granules. The PTEX machinery is responsible for the export of *Plasmodium* proteins into the host cytosol (de Koning-Ward et al., 2009), and GRA17 loss-of-function phenotypes can be rescued by *Plasmodium* EXP2 (Gold et al., 2015). Yet, intriguingly, GRA17 or GRA23 does not play a role in protein export. However, recently, it was discovered that a PTEX-independent pool of EXP2 exists at the PVM in *P. falciparum* that facilitates the exchange of soluble macromolecules across this membrane (Garten et al., 2018). Indeed, there is a correlation between EXP2 expression levels and the frequency with which the channel can be detected. Removal of 53 amino acids from the *C*-terminus of EXP2, a region that is abundant in acidic residues, gives rise to a PVM channel with an altered voltage response. Combined, these studies suggest that EXP2 not only gates the PVM channel, but also forms the actual nutrient channel through the PVM. Interestingly, pores are also found in the PVM of the liver stage parasites (Bano et al., 2007). As EXP2 is also expressed in the liver stage parasites (Vaughan et al., 2012), it may be performing a similar role, pointing toward a conserved mechanism in both lifecycle stages.

If the nutrient pore of *P. falciparum* exists in an open confirmation, the incorporation of EXP2 into membranes would need to be regulated to permit nutrient exchange only at the PVM. One candidate regulatory protein is EXP1, a PV-resident protein previously proposed to function as a glutathione S-transferase (Lisewski et al., 2014). EXP1 coimmunoprecipitates with EXP2 (Mesén-Ramírez et al., 2016) and is critical for PVM nutrient channel activity (Mesén-Ramírez et al., 2019). Conditional knockout of EXP1 using the dimerizable Cre system (Collins et al., 2013) led to slow growth phenotypes comparable to those resulting from amino acid starvation (Babbitt et al., 2012) or after conditional depletion of members of the RhopH complex (Counihan et al., 2017; Ito et al., 2017; Sherling et al., 2017). Compared with wild-type parasites, EXP1 knockout parasites showed a reduced frequency of channel detection by patch clamping, and transgenic parasites expressing limiting levels of EXP1 were hypersensitive to low levels of amino acids. These phenotypes may be attributable to altered PVM morphology and distribution of EXP2. EXP2 membrane association and protein export via PTEX did not appear to be affected (Mesén-Ramírez et al., 2019), indicating that EXP1 does not regulate the insertion of EXP2 into the membrane. Nessel et al. (2020) have also generated EXP1 knockdown parasites using the TetR-DOZI aptamer system (Ganesan et al., 2016) and similarly concluded that EXP1 is responsible for proper distribution of EXP2 at the PVM. However, as their knockout EXP1 parasites progressed further through the cell cycle than the EXP2 knockdown parasites, this led the authors to conclude that EXP1 does not contribute to EXP2-dependent transport activities. The differences between the findings of the two EXP1 studies may stem from the timing of EXP1 knockdown and when this impacts on PVM morphology. It is likely that disturbances to the PVM from EXP1 knockdown would also affect the localization/function of other PVM proteins that are critical for the parasite to complete the cell cycle, although this was not investigated.

RON3, a protein secreted from the rhoptry bulb and that localizes to the parasite periphery following invasion, has also been linked to PVM nutrient uptake. Parasites deficient in RON3 exhibit a similar phenotype to parasites deficient in EXP2 and fail to progress beyond the ring stages (Low et al., 2019). They are not only affected in their ability to import glucose, as they also fail to export proteins into the erythrocyte cytosol. However, RON3 is unlikely to be a direct interacting partner of nutrient pore EXP2 or PTEX EXP2; the BioID2 proximity ligase system fused to EXP2 did not reveal an interaction with RON3 (Mesén-Ramírez et al., 2016), and only few peptides were identified in a PTEX immunoprecipitation (de Koning-Ward et al., 2009), despite the fact that RON3 is a 263-kDa protein. Thus, it remains unclear how RON3 contributes to both nutrient uptake and protein export, and further studies are required to assess whether EXP2 localization or assembly is affected in RON3-deficient parasites.

## Transport of Nutrients Across the PPM

Once soluble macromolecules from the host have gained access to the PV, they can be then be transported across the PPM via an array of parasite transporters. A recent review by Martin (2020) has documented parasite transporters present at the different membranes in a parasitized erythrocyte, including the PPM, and so here we highlight only a subset for which there is experimental support for localization at the PPM. These include aquaglyceroporin (AQP), formate-lactate channel (FNT), putative copper channel, cationic amino acid transporter (NTP1), hexose transporter (HT1), putative amino acid transporter (AAT1, AAAP3), putative Mg^2+^ transporter, ATP: ADP antiporter (AAC1, AAC2), putative MCP1, folate–biopterin transporter (FT1, FT2), equilibrative nucleoside transporter (ENT, ENT4), Zn^2+^ and Fe^2+^ transporter (ZIPCO), the ABC transporters known as multidrug-resistant proteins (MDR1, MDR2, and MDR5) and multidrug-resistant associated proteins (MRP1 and MRP2), putative inorganic anion exchanger (SulP), Pi: Na^+^ symporter (PiT), putative lipid/sterol: H^+^ antiporter (NPC1R), V type H^+^ ATPase, and a variety of P-ATPases (ATP1, ATP2, ATP4, ATP8, CuTP, and GCa).

Not all of the transporters at the PPM are essential for parasite survival. Gene disruption studies (reviewed in Martin, 2020) and a large-scale mutagenesis screen (Zhang et al., 2018) have provided insight into which of these many transporters are essential and therefore could constitute potential antimalaria drug targets. Although the identity of the PPM transporter for pantothenate remains unknown, it is also a candidate drug target given the reliance on pantothenate uptake for parasite survival. The putative pantothenate transporter (PPT) was suggested to serve this role as it could complement the growth phenotype of a yeast mutant deficient in pantothenate transport (Augagneur et al., 2013). However, a study using rodent malaria parasites revealed that this transporter, which is also referred to as the putative metabolite transporter TFP1, is not expressed at the PPM in asexual blood stages and instead localizes to osmiophilic bodies in gametocytes (Kehrer et al., 2016). Moreover, the *tfp1* gene is dispensable (Hart et al., 2014; Kehrer et al., 2016; Zhang et al., 2018). Alternate pantothenate transporter candidates have been proposed, including the putative metabolite/drug transporter *umf* and monocarboxylate transporter *mcp1* or one of the parasite’s mitochondrial carrier superfamily proteins (Martin, 2020), but experimental evidence is lacking for any of these. Interestingly, the identity of the isoleucine PPM transporter, another potential antimalaria drug target due to the parasite’s requirement to take up isoleucine, is also unknown. Proposed candidates include the putative amino acid transporters AAT1 and AAAP3 (Martin, 2020), both of which were not mutable in *P. falciparum* (Zhang et al., 2018).

## Ingestion of Host Hemoglobin via Formation of the Cytostome

Unlike other microbes that source heme iron from hemoglobin through extraction from soluble and cell surface/wall receptors and subsequent transfer to cell wall and ABC transporters (Pishchany and Skaar, 2012), *P. falciparum* utilizes hemoglobin as a source of amino acids, which it instead acquires by endocytosing a large proportion of the host cell cytosol. This endocytosis process is non-selective, involving double-membrane invagination of both the PVM and the PPM, resulting in formation of a cytostome and potentially a larger structure known as a phagotroph (Aikawa et al., 1966; Slomianny et al., 1985; Slomianny, 1990; Elliott et al., 2008). The content of cytostomes has been assumed to be subsequently transported to the parasite’s digestive vacuole (DV); however, it is important to note that this has yet to be experimentally shown; consequently, the mechanism(s) by which hemoglobin and the host cell cytosol are delivered from endocytic structures to the DVs is yet to be resolved. We refer readers to a review by Spielmann et al. (2020) for proposed endocytic routes to the DV, which involve either the entire cytostome or only a portion pinching off and the subsequent transport to the DV, with potential involvement of intermediatory compartments. Digestion of hemoglobin occurs *en route* and within the DV (Abu Bakar et al., 2010), with the globin chains first cleaved from hemoglobin by aspartic proteases and cysteine endoproteases, and the liberated polypeptides then further digested by metalloproteases and series of aminopeptidases into oligopeptides and dipeptides to provide a source of amino acids for the parasite (Goldberg, 2005).

The molecular constituents that govern the uptake of the host cytosol by *P. falciparum* are also not resolved, largely because findings have either been conflicting or direct functional data have been lacking. We refer readers to two reviews that summarize the large body of work that has been undertaken to tease out potential molecular players in endocytosis of the host cell cytosol, with candidates including adapter protein-2 (AP-2), phosphatidylinositol-3-kinase, coronin, actin, dynamin, epidermal growth factor receptor substrate-15 (Eps15) homology domain (EHD) protein, soluble *N*-ethylmaleimide sensitive factor attachment protein (SNAP) receptor proteins (SNAREs), and Rab proteins (Spielmann et al., 2020; Xie et al., 2020). More recently, two studies from the Spielmann laboratory using genetic and proximity ligation approaches have shed further light on this process, revealing that components of a non-canonical endocytic apparatus are involved. In the first study, vacuolar protein sorting associated protein 4, which serves as an endolysosomal transport protein in other eukaryotes (Cowles et al., 1994; Piper et al., 1994), was shown to be required for host cell cytosol uptake (Jonscher et al., 2019). Then, a follow-up study examining the cellular function of Kelch 13 and its contribution to artemisinin resistance demonstrated that a Kelch-13 protein complex is also required for endocytosis of hemoglobin from the host cell (Birnbaum et al., 2020). This complex comprises Kelch 13, epidermal growth factor receptor substrate-15 (Eps15), ubiquitin carboxyl-terminal hydrolase (UBP1), AP-2, and a handful of other Kelch interacting proteins (Birnbaum et al., 2020). Unlike Kelch 13, which is only critical for the endocytosis of the host cell cytosol at ring stages, Eps15, UBP1, and AP-2 are required for endocytosis at both ring and trophozoite stages, such that inactivation of these proteins leads to a reduced transport of hemoglobin to the DV (Birnbaum et al., 2020). The identification of some of the molecular components of this endocytic pathway will now enable detailed mechanistic dissection of host cell cytosol uptake by the parasite.

## Concluding Remarks

Until recently, many of the molecular players involved in nutrient uptake by *Plasmodium* parasites have remained elusive, largely due to the difficulties associated with genetic manipulation of the *Plasmodium* genome and studying the function of essential genes (de Koning-Ward et al., 2015). However, the use of advanced molecular approaches to conditionally knock out or knock down genes and their products has enabled a better understanding of this process. Many of the genes identified in the pathways discussed in this review are essential to parasite survival, which highlights that blocking parasite nutrient acquisition from the host with antimalaria drugs is a worthwhile strategy to pursue. For drugs such as artemisinin that are activated by hemoglobin-derived heme and thus are dependent on the adequate uptake of hemoglobin by the parasite, understanding how this is achieved could inform approaches that could be used to antagonize drug resistance. Conversely, the molecular constituents of the hemoglobin uptake pathway could also be targeted with drugs, albeit not in combination with artemisinin or other heme-activated compounds. However, there are still some key knowledge gaps remaining. Functional evidence for CLAG3 serving as the channel-forming component of the NPP is lacking; if it does form the channel, what processes occur to drive insertion of CLAG3 into the membrane. If CLAG3 does not perform this role, what protein does, and is it parasite-derived? What function is served by the other CLAG proteins; are they required for the uptake of specific substrates? Why is the RhopH complex synthesized at the end of the cell cycle and secreted during invasion when the NPPs are not established for 15 h later, and what essential role do RhopH2 and RhopH3 play in NPP formation? What process ensures that EXP2 can form both a protein export channel and a nutrient channel, and what regulates its assembly into the PVM so that it does not prematurely form pores in membranes prior to this time? And is EXP2 the only nutrient channel at the PVM? What is the identity of the PPM transporters that are responsible for the uptake of isoleucine and pantothenate that are known to be essential to the parasite? These are just some of the questions that remain unanswered and are key to fully understanding the essential process of nutrient acquisition by *Plasmodium* parasites.

## Author Contributions

NC, JM, and TdK-W were responsible for writing and editing manuscript drafts and the final document. All authors contributed to the article and approved the submitted version.

## Conflict of Interest

The authors declare that the research was conducted in the absence of any commercial or financial relationships that could be construed as a potential conflict of interest.
